# Autophagy in chronic rhinosinusitis with or without nasal polyps

**DOI:** 10.3389/fcell.2024.1417735

**Published:** 2024-06-12

**Authors:** Jing Pei, Zhaoran Ding, Cheng Jiao, Ying Tao, Huifen Yang, Jing Li

**Affiliations:** ^1^ Department of Otolaryngology, Head and Neck Surgery, The Affiliated Jiangning Hospital With Nanjing Medical University, Nanjing, Jiangsu, China; ^2^ Department of Otorhinolaryngology Head and Neck Surgery, Northern Jiangsu People’s Hospital Affiliated to Yangzhou University, Yangzhou, Jiangsu, China; ^3^ Department of Blood Purification Center, Northern Jiangsu People’s Hospital Affiliated to Yangzhou University, Yangzhou, Jiangsu, China

**Keywords:** chronic rhinosinusitis, nasal polyps, autophagy, CRSsNP, CRSwNP

## Abstract

Basic research on chronic rhinosinusitis (CRS) has advanced significantly in the past two decades, yet a comprehensive understanding of its pathogenic mechanisms remains elusive. Concurrently, there is a growing interest among scientists in exploring the involvement of autophagy in various human diseases, including tumors and inflammatory conditions. While the role of autophagy in asthma has been extensively studied in airway inflammatory diseases, its significance in CRS with or without nasal polyps (NPs), a condition closely linked to asthma pathophysiology, has also garnered attention, albeit with conflicting findings across studies. This review delves into the role of autophagy in CRS, suggesting that modulating autophagy to regulate inflammatory responses could potentially serve as a novel therapeutic target.

## Introduction

**FIGURE 1:**
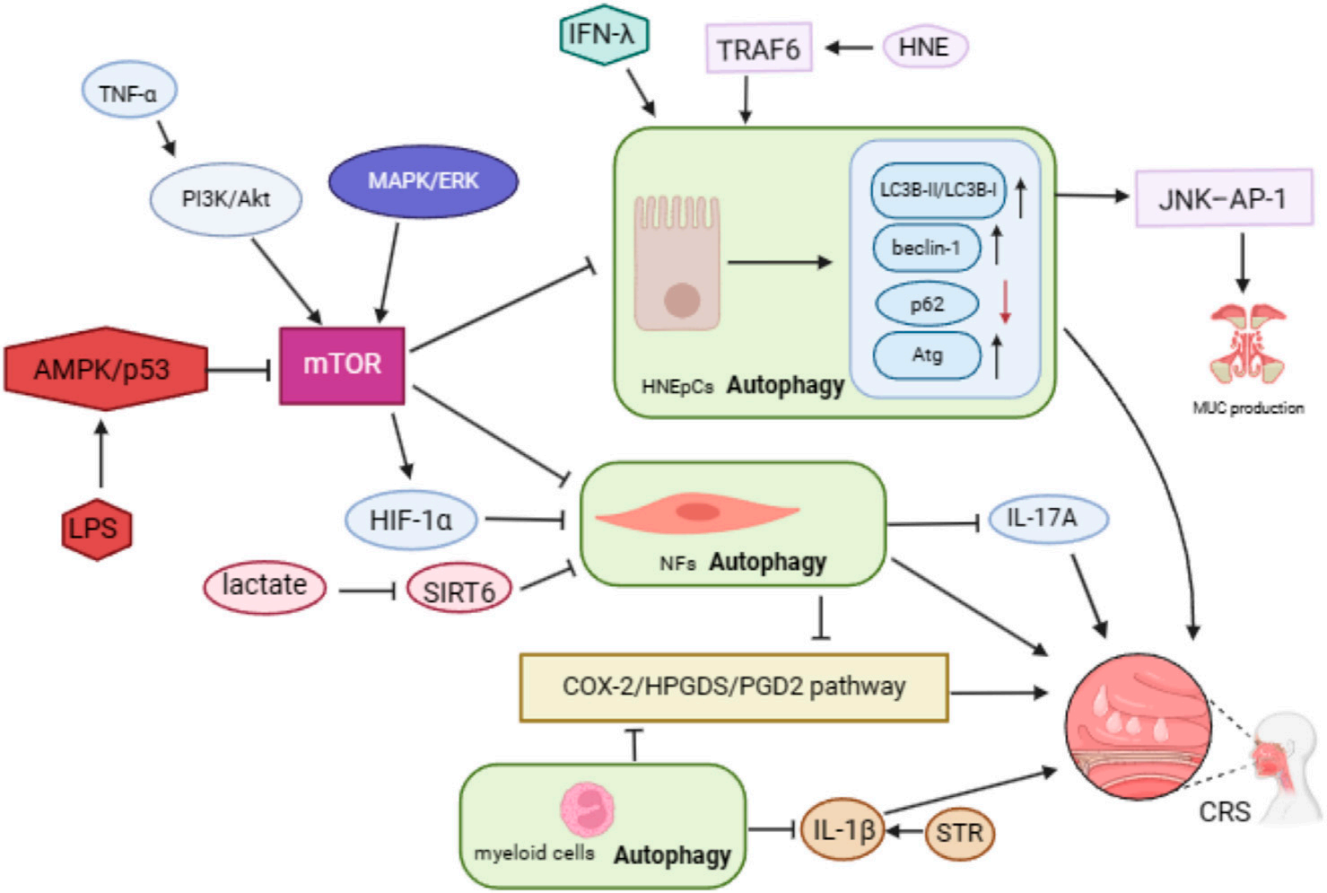
Mechanisms of autophagy in CRS.

Chronic rhinosinusitis (CRS) is a highly heterogeneous disease characterized by long-term and recurrent mucosal inflammation of the nasal cavity and sinuses, presenting symptoms such as nasal congestion, rhinorrhea, headache, facial pressure, and hyposmia (Subspecialty Group of Rhinology, Editorial Board of Chinese Journal of Otorhinolaryngology Head and Neck Surgery, and Subspecialty Group of Rhinology, Society of Otorhinolaryngology Head and Neck Surgery, Chinese Medical Association, 2019; Fokkens et al., 2020) (Figure 1). Nasal polyps (NPs) are structures resembling lychee fruits that form due to swelling and hyperplasia of the nasal mucosa after prolonged inflammatory stimulation. Epidemiological studies indicate that approximately one-third of CRS patients also have NPs, leading to the diagnosis of chronic rhinosinusitis with nasal polyps (CRSwNP) (Chee et al., 2022; Sedaghat et al., 2022; Starry et al., 2022). Pathological subtyping of CRSwNP based on the infiltration of inflammatory cells in the polyp tissue lacks uniform international criteria. Eosinophilic chronic rhinosinusitis with nasal polyp (eCRSwNP) is suggested to have a diagnostic criterion of eosinophils equal to or greater than 10 per high magnification field of view (Fokkens et al., 2020). Other pathological types, such as lymphocyte-type, plasma cell-type, neutrophilic-type, and mixed CRSwNP, are collectively referred to as non-eosinophilic chronic rhinosinusitis with nasal polyp (non-eCRSwNP) (Subspecialty Group of Rhinology, Editorial Board of Chinese Journal of Otorhinolaryngology Head and Neck Surgery, and Subspecialty Group of Rhinology, Society of Otorhinolaryngology Head and Neck Surgery, Chinese Medical Association, 2019). Patients with CRSwNP, particularly eCRSwNP, generally exhibit a poorer prognosis and higher recurrence rates compared to those with chronic rhinosinusitis without polyps (CRSsNP) (Ho et al., 2018; Cai et al., 2023). eCRSwNP is typically associated with Th2 inflammation, while non-eCRSwNP and CRSsNP are commonly linked to Th1 inflammation and mixed (Th1 or Th17) type inflammation (Lin et al., 2023; Yang et al., 2023). The mechanisms underlying CRS are complex and involve interactions between the host immune system, microbiota, and environmental factors (Subspecialty Group of Rhinology, Editorial Board of Chinese Journal of Otorhinolaryngology Head and Neck Surgery, and Subspecialty Group of Rhinology, Society of Otorhinolaryngology Head and Neck Surgery, Chinese Medical Association, 2019; Fokkens et al., 2020; Goulioumis et al., 2023). Currently, targeted drugs focusing on Th2 type inflammatory factors like anti-IL-4 and anti-IL-5 have received approval in certain countries for treating CRSwNP. However, their limited use is attributed to high costs, specific indications, and uncertain long-term efficacy (Bachert et al., 2020). Thus, exploring new targets or pathways to regulate CRSwNP progression could lead to more efficient and cost-effective treatment options in clinical practice.

Autophagy plays a crucial role in defending against external pathogenic microorganisms, removing aging and harmful substances from cells, and maintaining cellular homeostasis and function. It accomplishes this by delivering cytoplasmic components to lysosomes for degradation and amino acid recycling through macroautophagy, chaperone-mediated autophagy, and microautophagy pathways (Esclatine et al., 2009; Feng et al., 2014; Munz, 2014). Dysregulation of autophagy has been linked to various diseases such as cancer, neurodegenerative diseases, reproductive system diseases, and inflammatory diseases (Chandrasekaran et al., 2023; Debnath et al., 2023; Dong et al., 2023; Kirat et al., 2023). Recent research has highlighted the importance of autophagy in the immune system, where it aids in pathogen removal, immune cell differentiation, antigen presentation, and regulation of inflammatory responses (Germic et al., 2019; Munz, 2021; Shariq et al., 2023). The connection between airway inflammation and autophagy has been a focus of investigation, particularly in the context of asthma (Lee and Kim, 2019; Painter et al., 2020; Theofani and Xanthou, 2021; Wang et al., 2021). Autophagy has been shown to have both protective and detrimental effects in allergic asthma, with a balancing role to prevent excessive lung tissue damage while mounting a protective anti-pathogen response (Theofani and Xanthou, 2021). Moreover, autophagy has been identified as a key regulator of asthma airway remodeling, influencing airway wall thickening and rigidity, and can also contribute to mitigating fibrosis by degrading extracellular matrix (ECM) proteins (Herrera et al., 2018).

Due to the close relationship and similarities between upper and lower respiratory tract inflammation, otolaryngologists have recently started investigating the potential role of autophagy in CRS. As research progresses, there have been some controversies regarding the involvement of autophagy in CRS. This paper aims to summarize the existing studies on this topic in hopes of guiding future research.

## Classical markers and pathways of autophagy

Autophagy, classified as a type II programmed cell death, is a crucial self-degradation process essential for energy maintenance during development and in response to stress. This process can be either selective or non-selective (Dikic and Elazar, 2018). The core of autophagy lies in intracellular membrane rearrangement, which occurs in four main stages: extension of a phagocytic bubble, formation of isolated membranes and autophagosomes, fusion of autophagosomes with lysosomes, and cleavage of autophagosomes (Li et al., 2020; McAlinden et al., 2019). Various autophagy-related proteins, synthesized by autophagy-related genes (ATG), play a role in regulating and controlling the different stages of autophagy formation (Li et al., 2020).

LC3, also known as MAP1LC3, serves as a well-established autophagy marker throughout the autophagic process. In mammals, LC3 exists in three types: LC3A, LC3B, and LC3C, with LC3B being the most commonly utilized. When the LC3 protein is synthesized, the C-terminal 5-peptide is sheared off by Atg4 to produce LC3-I. During autophagy, LC3-I is modified and processed by a ubiquitin-like system including Atg7 and Atg3, which couples with phosphatidylethanolamine (PE) to form LC3-II and localize to the autophagosome’s inner and outer membranes. Upon fusion of autophagosome and lysosome, LC3-II on the outer membrane is cleaved by Atg4 to produce LC3-I for recycling; LC3-II on the inner membrane is degraded by lysosomal enzymes, resulting in low LC3 content in autophagic lysosomes (Kiriyama and Nochi, 2015). The ratio of LC3 II/LC3I is a widely used indicator of autophagic activity, where a higher ratio corresponds to increased autophagic activity.

In addition to LC3, changes in the expression of other autophagic substrates can also be used to monitor autophagic flow. Among these, p62 is commonly utilized. During autophagosome formation, p62 acts as a bridge linking LC3 and polyubiquitinated proteins, being selectively enclosed within autophagosomes. Subsequently, it undergoes degradation by proteohydrolases in autophagic lysosomes, leading to a negative correlation between the expression of the p62 protein and autophagic activity (Ichimura et al., 2008). Beclin-1, a key regulatory protein of autophagy encoded by the BECN1 gene, participates in the formation of autophagosome membranes. Therefore, elevated levels of Beclin-1 indicate heightened autophagic activity (Xu and Qin, 2019).

Several autophagy-related pathways have been identified, with notable classical pathways including the phosphoinositide 3-kinase (PI3K)/protein kinase B (Akt) and Mitogen-Activated Protein Kinases (MAPK)/Extracellular Signal-Regulated Kinase (ERK) pathways that activate mammalian target of rapamycin protein (mTOR), as well as the Adenosine Monophosphate Activated Protein Kinase (AMPK) and p53 pathways that inhibit mTOR (Yang and Klionsky, 2010; Yu et al., 2018; Ba et al., 2019). mTOR serves as a crucial negative regulator of cellular autophagy, being activated in nutrient-sufficient conditions to suppress autophagy and inhibited during starvation or stress to promote autophagy (Wang and Zhang, 2019). ULK, possessing serine/threonine kinase activity, is the core protein of the autophagy signaling pathway. The ULK1 complex acts as a link between upstream mTOR and AMPK and downstream autophagosome formation. Activation of AMPK or inhibition of mTOR phosphorylates ULK1, thereby enhancing autophagy (Kim et al., 2011).

## Autophagy phenomenon in tissues of CRS patients

Autophagy phenomenon in tissues of CRS patients remains a topic of debate in the scientific community (Table 1). While some studies suggest autophagic insufficiency or excess in CRS, the majority lean towards a deficiency in autophagy. For example, Western blot (WB) analysis indicated a significant decrease in LC3 expression and an increase in p-Akt and p-mTOR in nasal tissue specimens of CRS patients compared to normal nasal mucosae. Immunohistochemical (IHC) analysis further supported these findings, showing weaker LC3 and stronger p-mTOR staining in nasal polyps (Chen et al., 2015). Analogously, Wang L F. et al. analyzed the nasal mucosa from six CRSsNP patients and the NPs obtained from five CRSwNP patients via WB, showing that low LC3 but high cyclooxygenase-2 (COX-2) expression in NPs. IHC results showed that most NPs (17/23) had negative to weak LC3 staining intensity, while more than half of them (12/23) had moderate to strong COX-2 staining intensity. Besides, an inverse relationship between LC3 and COX-2 expression was identified using Kappa statistic, suggesting that insufficient autophagy may have contributed to increased inflammation (Wang et al., 2015). Compared with normal inferior turbinate mucosa (*n* = 20), the expression of LC3B and Beclin-1 were deficient and p62 was stronger in specimens of CRSwNP (*n* = 50) both in IHC and reverse transcription-polymerase chain reaction (RT-PCR) results (Qi et al., 2016). IHC with 35 nasal tissue sections also revealed that LC3 immunoreactivity was decreased in the NPs compared to the mucosa from patients with compensatory turbinate hypertrophy. In their IHC results, mTOR also had stronger positive signal (Simsek et al., 2021). Overall, the evidence points towards a deficiency in autophagy in CRS patients, as supported by various molecular analyses and IHC results. The levels of p-Akt and PI3K proteins were significantly higher in NPs compared to nasal tissues from sinus cyst patients, indicating a potential downregulation of autophagy in CRSwNP patients. Additionally, increased myeloperoxidase (MPO) activity, mRNA levels of hypoxia-inducible factor-1α (HIF-1α), and IL-17A in NPs suggest a link between hypoxia-induced neutrophil inflammation and inadequate autophagy (Cheng et al., 2022). Wang et al. (2022)’s study comparing various protein markers in different CRSwNP groups revealed an activated Akt-mTOR pathway and decreased levels of beclin-1 and LC3II in CRSwNP patients, with no significant differences between eCRSwNP and non-eCRSwNP groups. Interestingly, p62 levels did not vary significantly between groups in IHC experiments but were notably reduced in control tissues compared to eCRSwNP tissues in WB experiments. In addition, Transmission Electron Microscopy (TEM) showed less frequent formation of autophagosomes in both CRSwNP endotypes compared to control tissues. The study’s findings suggest a deficiency in autophagy in CRSwNP patients, regardless of endotype. Furthermore, they also investigated mitophagy-related markers (PINK1, BNIP3, and FUNDC1) with WB and tissue remodeling conditions using Masson’s trichrome (MT) and Periodic acid–Schiff Alcian blue (PAS–AB) staining, and they found that the levels of PINK1 and BNIP3 were all negatively correlated with collagen deposition in non-eCRSwNP patients, suggesting that mitophagy may play an important role in tissue remodeling in patients with non-eCRSwNP (Wang et al., 2022).

**TABLE 1:** Autophagy phenomenon in tissues of CRS patients.

Viewpoint	Reference	Upregulated genes or proteins	Downregulated genes or proteins	Tissue specimens
Autophagy deficiency	Chen et al. (2015)	p-Akt, p-mTOR	LC3	NPs vs. nasal mucosa from NCs
	Wang et al. (2015)	COX-2	LC3	NPs vs. nasal mucosa from CRSsNP
	Qi et al. (2016)	p62	LC3B, beclin-1	NPs vs. nasal mucosa from NCs
	Simsek et al. (2021)	mTOR	LC3	NPs vs. nasal mucosa from patients with compensatory turbinate hypertrophy
	Cheng et al. (2022)	p-Akt, PI3K, MPO, HIF-1α, IL17		NPs vs. nasal mucosa from sinus cysts patients
	Wang et al. (2022)	p-Akt, p-mTOR	beclin-1, LC3II, PINK1, BNIP3, FUNDC1	NPs (eCRSwNP and non-eCRSwNP) vs. nasal mucosa from NCs
Autophagy excess	Shun et al. (2016)	LC3II, beclin		NPs vs. nasal mucosa from NCs
	Wang et al. (2017a)	LC3B-II, beclin-1, Atg3, Atg5, Atg7, p62		NPs (eCRSwNP and non-eCRSwNP) vs. nasal mucosa from NCs
	Wang et al. (2017b)	LC3B		UT from CRSsNP and NCs
	Ye et al. (2019)	ATG5, BECN1, LC3B, HNE, MUC5AC		NPs vs. UT from CRSsNP vs. nasal mucosa from NCs
	Zhang et al. (2022)	BECN1, ATG5, LC3B, HNE, MUC5AC		NPs vs. nasal mucosa from NCs

NPs, nasal polyps; NCs, normal controls; UT, uncinate tissues.

Contrary to some beliefs, studies have shown that enhanced autophagy is present in patients with chronic rhinosinusitis (CRS). For example, WB results revealed increased expression of LC3II in 20 NPs specimens compared to 5 specimens of normal nasal mucosa, with IHC also indicating enhanced beclin levels in NPs, suggesting autophagic excess (Shun et al., 2016). Another study found elevated expression of autophagic proteins (LC3B-II, beclin-1, Atg3, Atg5, Atg7, and p62) and increased autophagic vacuole formation in both eCRSwNP and non-eCRSwNP tissues compared to normal controls (Wang et al., 2017a). Wang et al. (2017b) further investigated LC3B expression levels in uncinate tissues (UT) from CRSsNP patients and normal controls, revealing LC3B overexpression in CRSsNP patients. Ye et al. (2019) compared autophagic activity in polyp tissue from CRSwNP patients, UT from CRSsNP patients, and nasal mucosa from patients with simple septal deviation, showing increased mRNA levels of ATG5 and BECN1 in CRS, particularly in CRSwNP. Additionally, IHC analysis confirmed autophagic upregulation in CRS by comparing LC3B expression at the protein level in the three groups. Both human neutrophil elastase (HNE) and mucin 5AC (MUC5AC) were found to be upregulated simultaneously, suggesting that autophagy activation may promote HNE-induced mucus production (Ye et al., 2019). This was supported by another study showing increased mRNA levels of BECN1, ATG5, HNE, and MUC5AC by RT-PCR, as well as stronger positive signals of LC3B, HNE, and MUC5AC proteins in CRSwNP tissue samples compared to controls by IHC (Zhang et al., 2022).

## Autophagy in cellular experiments and animal models

Autophagy marker levels in nasal mucosa or polyp samples from patients with CRS have been used to investigate the role of autophagy in the development of CRS and its underlying mechanisms (Table 2). Various cellular and animal experiments have been conducted by researchers to further understand this relationship.

**TABLE 2:** Autophagy in cellular experiments and animal models.

Viewpoint	Reference	Cellular experiment	Animal experiment	Inflammation mechanism or pathways
Autophagy deficiency	Wang et al. (2015)	NFs		COX-2 was negatively regulated by autophagy
	Choi et al. (2018)		eCRS mouse model with myeloid cell specific Atg7 deletion	The expression of COX-2, HPGDS and PGD2 in mRNA and protein level all significantly increased in Atg7 deletion eCRS mice. Autophagy deficiency in myeloid cells exacerbates eosinophilic inflammation in CRS and it is IL-1 dependent
	Lee et al. (2022)		eCRS mouse model with myeloid cell specific Atg7 deletion	Sweet taste receptor agonists attenuate macrophage IL-1β expression and eosinophilic inflammation linked to autophagy deficiency in myeloid cells
	Cheng et al. (2022)	NFs		The PI3K/Akt/HIF-1α pathway regulated the IL-17A-related inflammation in CRSwNP
Autophagy excess	Shun et al. (2016)	NFs		Exogenous lactate can reverse the suppressive effect of SIRT6 on autophagy
	Wang et al. (2017b)	HNEpCs		LPS induces autophagy by targeting the AMPK-mTOR pathway but not PI3K/Akt
	Wang et al. (2017a)	BEAS-2B cells and HNEpCs		IFN-λ, but not IL-4, IL-13, or IL-17A, simultaneously enhanced LC3B-II and p62 levels
	Ye et al. (2019)	HNEpCs		Autophagy is indispensable for HNE-induced activation of the JNK–AP-1 signaling pathway, and it subsequently promote excessive MUC production
	Zhang et al. (2022)	HNEpCs		HNE/TRAF6/autophagy axis might be an important pathogenic mechanism for MUC5AC hyperproduction in CRSwNP

NFs, nasal fibroblasts; HNEpCs, human nasal epithelial cells.

Firstly, we summarize those studies in which CRS suffered from autophagy deficiency. Nasal fibroblasts (NFs) were isolated and cultured from NPs of CRSwNP patients (NPFs) and nasal mucosa of CRSsNP patients (NMFs). Autophagy induction through starvation and LC3 overexpression in both NFs led to a decrease in COX-2 expression, a common indicator of inflammation. Conversely, inhibiting autophagy with 3-methyladenine (3-MA) increased COX-2 expression. Interestingly, treatment with pro-inflammatory cytokines IL-1β and TNF-α did not significantly alter LC3 expression but did induce COX-2 expression in both NPFs and NMFs (Wang et al., 2015). These findings suggest that the chronic mucosal inflammation seen in CRSwNP may be due to a persistent autophagy deficiency, and manipulating autophagy could offer a promising therapeutic approach for CRSwNP. Choi et al. (2018) investigated the impact of impaired autophagy on eosinophilic inflammation in eosinophilic chronic rhinosinusitis (eCRS) mouse models through myeloid cell-specific deletion of Atg7. Their study revealed that autophagy deficiency in myeloid cells exacerbated eosinophilia, mucosal thickening, and epithelial hyperplasia in eCRS mice. While the counts of leukocytes, neutrophils, lymphocytes, basophils, and monocytes remained unchanged, there was a significant increase in eosinophil numbers in the blood of eCRS mice with impaired autophagy. Additionally, they explored the potential mechanisms and observed a substantial increase in the expression of COX-2, hematopoietic prostaglandin D synthase (HPGDS), and prostaglandin D2 (PGD2) at both mRNA and protein levels in Atg7 deletion eCRS mice. Moreover, the researchers demonstrated that blockade of the IL-1 receptor could alleviate eosinophilic inflammation, suggesting an IL-1 dependent pathway in eCRS due to autophagy deficiency. In a separate study, Lee et al. (2022) discovered that the anti-inflammatory effects of sweet taste receptor (STR) agonists, particularly trehalose, significantly mitigated eosinophilia, and disease pathogenesis in eCRS mice with autophagy deficiency in myeloid cells. Their mechanistic investigation revealed the involvement of T1R3 in reducing macrophage IL-1β production and eosinophilia in CRS, which was supported by genetic manipulation of T1R3 expression in macrophages and treatment with the T1R3 antagonist gurmarin. This research highlighted a previously overlooked anti-inflammatory target, offering a potential strategy for modulating autophagy in the treatment of eCRS (Lee et al., 2022). NFs isolated from CRSwNP patients were cultured to investigate the autophagy pathway. Upon stimulation with Tumor Necrosis Factor-alpha (TNF-α), levels of PI3K, p-AKT, HIF-1α, and IL-17A significantly increased in the fibroblasts. However, upon application of Wortmannin (a selective PI3K inhibitor), these indicators decreased significantly. This suggests that the PI3K/Akt/HIF-1α pathway may regulate IL-17A-related inflammation in CRSwNP (Cheng et al., 2022).

Several studies have indicated an increase in autophagy in CRS. Shun et al. (2016) discovered that Sirtuin 6 (SIRT6) expression was decreased while beclin was upregulated in NPFs. Additionally, under hypoxic conditions, the levels of LC3II in NPFs increased in a time-dependent manner, peaking at 12 h. Overexpression of SIRT6 was found to suppress hypoxia induced LC3II production in NPFs; however, the addition of exogenous lactate could reverse this suppressive effect on autophagy. In another study, human Nasal Epithelial Cells (HNEpCs) treated with lipopolysaccharide (LPS)—a common pathogenic element in CRS - showed dose- and time-dependent induction of autophagy. This was evidenced by increased levels of LC3B-II, decreased levels of p62 as observed through WB, and the presence of autophagosomes observed through TEM. Further exploration into the mechanism of autophagy involved Western blot analysis of proteins in the PI3K/Akt and AMPK pathways, revealing no significant changes in p-Akt, Akt, AMPK, and mTOR, but a notable increase in p-AMPK and decrease in p-mTOR. Treatment of HNEpC with Compound C—an AMPK inhibitor—after LPS exposure resulted in the inhibition of autophagy, indicating that LPS induces autophagy in HNEpC by targeting the AMPK-mTOR pathway (Wang et al., 2017b). Wang et al. (2017a) found that IFN-λ increased levels of autophagic proteins in both eCRSwNP and non-eCRSwNP tissues. Through cellular experiments, they discovered that IFN-λ enhanced LC3B-II and p62 levels in BEAS-2B cells and HNEpCs, while inhibitors like bafilomycin A1 led to increased levels of autophagic proteins. This suggests that IFN-λ induces activated but insufficient autophagy in CRSwNP. Autophagy activity was also found to be involved in regulating mucus secretion, with HNE inducing MUC5AC expression via the AP-1 pathway in primary HNEpCs. Inhibition of autophagy pathways and TRAF6 were shown to alleviate hypersecretion of MUC5AC, indicating the potential importance of the HNE/TRAF6/autophagy axis in CRSwNP pathogenesis (Zhang et al., 2022).

## Transcriptome sequencing

Recent transcriptome sequencing has identified differentially expressed genes related to the autophagy pathway in CRS, suggesting a potential role of autophagy in the pathogenesis of CRS. For instance, Xiong et al. (2020) conducted transcriptome analysis and found that epithelial cell injury, autophagy, and the mTOR pathway (hsa04140 and hsa04150) may play a role in the development of non-eCRSwNP. Furthermore, bulk RNA sequencing and single cell RNA-seq datasets of epithelial cells (EpCs) from CRS patients revealed upregulation of mTOR complex 1 (mTORC1) activity and glycolytic activity in CRSwNP. *Ex vivo* inhibition experiments suggested that mTOR plays a critical role in EpC production of CXCL8, IL-33, and CXCL2. Enhanced mTORC1 signaling in CRSwNP EpCs was found to be associated with glycolysis, rather than mTORC2 (a typical PI3K/Akt/mTOR signature gene). Epithelial glycolysis can be induced by Th2 and Th17 cytokines *in vitro* and is correlated with Th2 cytokine response *in vivo*. These findings imply that CRSwNP may exhibit autophagy deficiency, with mTORC1 playing a significant role in its pathogenesis, potentially linked to glycolytic activity (Huang et al., 2023). Additionally, bulk and single cell RNA-seq data highlighted the importance of Migration Inhibitory Factor (MIF) and Transforming Growth Factor-Beta (TGF-β) pathways in mediating the effects of mitophagy in CRS, particularly emphasizing the role of MIF (Zhou et al., 2023).

## Concluding remarks and future perspectives

From the overview above, autophagy displays both protective and detrimental effects on CRS. We speculate that autophagy is enhanced in the early stages of disease in CRS to remove cellular damage caused by pathogens or allergens to maintain organismal homeostasis, but as the damage persists, autophagy will be depleted and exhibit autophagic insufficiency. Current research leans towards autophagy deficiency in CRS, particularly in CRSwNP. However, the limited literature, small sample sizes in clinical studies, and scarcity of animal experiments highlight the need for further investigation. Studies have mainly focused on NFs from NPs tissues or control mucosa, as well as HNEpCs from healthy individuals, with conflicting findings on autophagy regulation. Most of the literature favors the presence of autophagy upregulation in HNEpCs and autophagy downregulation in NFs (Shun et al., 2016; Wang et al., 2017a; Wang et al., 2017b; Ye et al., 2019; Zhang et al., 2022). However, there is an exception which showed autophagy upregulation in NFs (Shun et al., 2016). Future *in vitro* experiments using both cell types could shed light on the differences in autophagy levels and their impact on CRS progression. The review focused on eCRS models, leaving room for further exploration of autophagy differences in non-eCRS models (Choi et al., 2018; Lee et al., 2022). Non-type 2 inflammation in CRS, represented by LPS, IFN-λ, and HNE-stimulated HNEpCs, often shows autophagy excess involving AMPK-mTOR, JNK–AP-1, and HNE-TRAF6 pathways (Wang et al., 2017a; Wang et al., 2017b; Ye et al., 2019). On the other hand, the PI3K/Akt/HIF-1α and COX-2/HPGDS/PGD2 pathway tends to regulate type-2 inflammation in CRS, leading to autophagy deficiency (Choi et al., 2018; Cheng et al., 2022). Interestingly, no significant differences in autophagy marker expression were observed between eCRSwNP and non-eCRSwNP tissue samples, although further confirmation with larger sample sizes is warranted (Wang et al., 2017a; Wang et al., 2022). The dual role of autophagy in CRS may vary among different cell types, as seen in lung inflammation. Myeloid lineage cells (especially neutrophils and eosinophils) and lymphoid lineage cells (e.g., CD4^+^, CD8^+^, ILC2, and B-cells) can enhance lung inflammation through autophagy modulation in distinct pathways, while epithelial cells and fibroblasts can influence lung remodeling via autophagy regulation (Painter et al., 2020). As current *in vitro* studies mainly focus on non-immune cells (HNEpCs and NFs), additional experiments are needed to understand the changes in autophagy levels in immune cells of CRS. The development of personalized autophagy modulators tailored to specific cell types and pathogenesis holds promise for future therapeutic interventions.

A recent study found that offspring of female mice exposed to OVA may reduce Th2 inflammation by enhancing autophagy in mast cells when exposed to OVA (Kim et al., 2021). Overexpression of Orosomucoid-like-3 (ORMDL3) increased the expression of SERCA2b, activating transcription factor 6 (ATF6), Beclin-1, and LC3BII. Conversely, inhibiting autophagy or knocking down ATF6 reversed the decrease in anaphylactic reaction caused by ORMDL3 overexpression (Li et al., 2021). Autophagy has also been linked to epithelial mesenchymal transition (EMT) and epigenetic changes in the fields of oncology, lung diseases, and diabetes (Ranieri et al., 2021; Zeng et al., 2021; Gundamaraju et al., 2022; Yu et al., 2024). Both EMT and epigenetic changes play roles in the pathogenesis of CRS (Li et al., 2022; Wang et al., 2023). These findings provide valuable insights for future research on CRS, highlighting the importance of further exploring the role of autophagy in this condition. While the current study is not without controversy, it sheds light on potential new therapeutic approaches for CRS by targeting autophagy.

## Figures and Tables

**Table:** 

**3-Ma**	3-methyladenine
**Akt**	protein kinase B
**AMPK**	Adenosine Monophosphate Activated Protein Kinase
**ATF6**	activating transcription factor 6
**ATG**	autophagy-related genes
**COX-2**	cyclooxygenase-2
**CRS**	Chronic rhinosinusitis
**CRSsNP**	chronic rhinosinusitis without polyps
**CRSwNP**	chronic rhinosinusitis with nasal polyp
**ECM**	extracellular matrix
**eCRS**	eosinophilic chronic rhinosinusitis
**eCRSwNP**	eosinophilic chronic rhinosinusitis with nasal polyp
**EMT**	epithelial mesenchymal transition
**EpCs**	epithelial cells
**ERK**	Extracellular Signal-Regulated Kinase
**HIF-1α**	hypoxia-inducible factor-1α
**HNE**	human neutrophil elastase
**HNEpC**	Human Nasal Epithelial Cells
**HPGDS**	hematopoietic prostaglandin D synthase
**IFN-λ**	interferon-λ
**IHC**	Immunohistochemical
**LPS**	lipopolysaccharide
**MAPK**	Mitogen Activated Protein Kinases
**MIF**	Migration Inhibitory Factor
**MPO**	myeloperoxidase
**MT**	Masson’s trichrome
**mTOR**	mammalian target of rapamycin protein
**mTORC**	mTOR complex
**MUC5AC**	mucin 5AC
**NFs**	Nasal fibroblasts
**non-eCRSwNP**	non-eosinophilic chronic rhinosinusitis with nasal polyp
**NPs**	nasal polyps
**ORMDL3**	Orosomucoid-like-3
**PAS–AB**	Periodic acid–Schiff Alcian blue
**PE**	phosphatidylethanolamine
**PGD2**	prostaglandin D2
**PI3K**	phosphoinositide 3-kinase
**RT-PCR**	reverse transcription-polymerase chain reaction
**scRNA-seq**	single cell RNA-seq
**SIRT6**	Sirtuin 6
**STR**	sweet taste receptor
**TEM**	Transmission Electron Microscopy
**TGF-β**	Transforming growth factor- beta
**TNF-α**	Tumor Necrosis Factor- alpha
**TRAF6**	TNF receptor-associated factor 6
**UT**	uncinate tissues
**WB**	Western blot
